# Stakeholder priorities for sustaining operations and maintenance of school sanitation facilities in Kampala City, Uganda

**DOI:** 10.1186/s12982-026-02148-x

**Published:** 2026-05-29

**Authors:** Jude Zziwa Byansi, Swaib Semiyaga, Alex Yasoni Katukiza, Najib Lukooya Bateganya, Angella Mercy Nakaye, Frank Kansiime, Robinah Nakawunde Kulabako

**Affiliations:** 1 Department of Civil and Environmental Engineering, College of Engineering, Design, Art and Technology, Makerere University, P. O. Box 7062, Kampala, Uganda; 2 Mulago Specialised Women and Neonatal Hospital, P.O. Box 22081, Kampala, Uganda; 3 Department of Environmental Management, College of Agricultural and Environmental Sciences, Makerere University, P. O. Box 7062, Kampala, Uganda

**Keywords:** School sanitation, Operation and maintenance, Analytic hierarchy process, SWING method, Sustainability

## Abstract

**Introduction:**

Sustaining sanitation services in schools in low resource settings requires effective operation and maintenance (O&M), yet the relative influence of management on service reliability remains poorly understood.

**Methods:**

This study employed a participatory multi-criteria decision-analysis (MCDA) design to identify stakeholder-derived priorities for O&M of sanitation facilities in Kampala schools. The Analytic Hierarchy Process (AHP) was used to weigh domains, and the SWING method was used to prioritise indicators. Stakeholders were selected using purposive sampling techniques. Participants included practitioners from civil society organisations involved in school sanitation programmes, and regulators from relevant ministries and Kampala Capital City Authority (KCCA). The sample also included researchers who had published on sanitation in Kampala, as well as Head Teachers and Sanitation Teachers from the study schools. Thirty five stakeholders attended a facilitated workshop and completed structured AHP and SWING weighting exercises. The data collection instruments were adapted from standard MCDA templates and customised for the school sanitation O&M context. Their validity was strengthened through expert review, pre-testing, and structured participant orientation. AHP consistency ratio checks were also conducted, and all responses met the required threshold (CR ≤ 0.1). Bootstrap confidence intervals were generated to assess robustness.

**Results:**

Service planning (0.234), resource management (0.199), and facility design standards (0.193) were ranked as the most influential domains. Governance (0.166), service delivery (0.137), and monitoring and evaluation (M&E) (0.071) were assigned lower weights. The highest priority indicators were the presence of an O&M plan, a dedicated budget line, clear roles and responsibilities, effective budget implementation, and reliable water supply.

**Conclusions and recommendations:**

These findings show that sustained sanitation service provision depends more on effective planning, predictable financing, accountability, and reliable inputs than solely on infrastructure. It is therefore recommended that schools sanitation programmes prioritize strengthening O&M planning, budgeting, and M&E systems to improve sanitation service reliability in resource-constrained school settings.

**Graphical Abstract:**

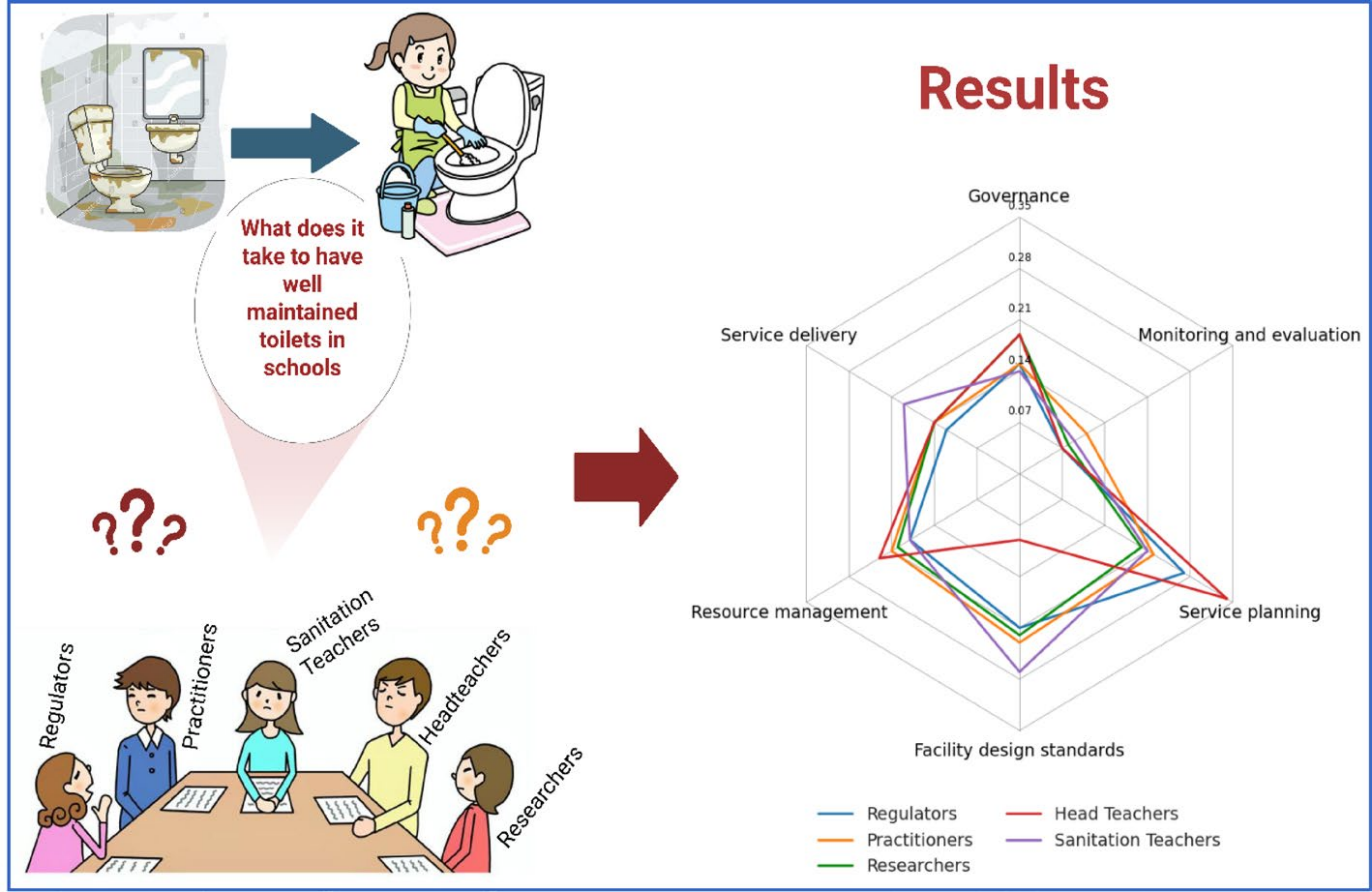

**Supplementary Information:**

The online version contains supplementary material available at 10.1186/s12982-026-02148-x.

## Highlights

The study incorporates perspectives from regulators, practitioners, Head Teachers, Sanitation Teachers, and researchers.

Management domains ranked highest: planning, resources, design standards.

Monitoring and evaluation (M&E) received the lowest weights across groups.

Top indicators: O&M plan, budget line, role and responsibility clarity, budget execution, water supply.

No significant differences in domain weights across stakeholder groups.

AHP and SWING produced a practical, stakeholder informed priority framework.

**Supplementary Information:**

The online version contains supplementary material available at 10.1186/s12982-026-02148-x.

## Introduction

Sanitation service provision in schools requires not only access to infrastructure but also active involvement of stakeholders who plan, finance, operate, and oversee the facilities [48]. Studies across diverse low- and middle-income countries (LMICs) settings show that school sanitation systems often fail due to weak coordination among stakeholders, such as ministries, local authorities, school leaders, teachers and community partners. The coordination gaps result in irregular or poor maintenance, limited consumable supplies, and inadequate monitoring, even where improved facilities exist [7, 18, 49]. Globally, about 205 million pupils attend schools that do not provide a basic sanitation service, even though improved sanitation technologies may be present WHO/UNICEF, [74]. Where schools have improved sanitation technologies but fail to achieve basic sanitation service levels, this commonly reflects weaknesses in operation and maintenance related to functionality, accessibility, sex segregation, and privacy rather than the absence of infrastructure. In Sub-Saharan Africa, including East Africa, about half of schools fail to meet basic sanitation service criteria, reflecting persistent challenges in sustaining functionality, accessibility, and privacy of sanitation facilities. Poorly maintained sanitation facilities expose pupils to infection, increase absenteeism, and disrupt learning, which underscores the role of effective operation and maintenance (O&M) in sustaining public health and educational benefits. Evidence from school water sanitation and hygiene (WASH) programmes shows that aligned stakeholder priorities strengthen accountability and direct O&M resources toward the activities most critical for service quality, improving facility functionality and reliability [17, 20].

In Uganda, about 23% of schools operate at a limited sanitation service level, a condition that is associated with deficiencies in O&M of sanitation facilities [13]; WHO/UNICEF, [74]. The responsibility for school sanitation in Uganda is distributed across several ministries and local government structures, creating overlapping mandates and inconsistent support for routine O&M. In Kampala City, 62% of schools operate at a limited sanitation service level, reflecting challenges in sustaining O&M of sanitation facilities [13]. Rising pupil enrolment, coupled with uneven institutional capacity across public and private schools, makes coordinated service provision difficult and weakens sustained sanitation management in Kampala [13, 37]. This complexity underscores the need to clearly identify the domains that support effective O&M of sanitation facilities, including planning, resource availability, cleaning and repair routines, user engagement, and monitoring and evaluation (M&E).

Service outcomes are strongly influenced by how the interests and actions of different actors are recognised, aligned, and managed [11]. With respect to school sanitation facilities, this implies that sustained O&M depends on how stakeholders: (1) understand their roles, (2) coordinate their actions, and (3) prioritise the tasks required to keep the facilities functional. When stakeholders align their priorities, resources for budgeting, consumables, cleaning, repairs, and M&E are more consistently directed toward the most influential aspects of service provision. This alignment enhances facility functionality, supports more reliable cleanliness and hygiene, and contributes to higher levels of pupil satisfaction with school sanitation services [24, 43].

Several studies have identified conditions such as resource availability, accountability structures, information systems, facility standards, community engagement, and strong local leadership essential for long-term service delivery in schools [17, 18, 21, 49]. While these studies describe the requirements for sustained service provision, there is limited empirical evidence on how different stakeholder groups prioritise the identified conditions in schools. Without such insights, there is a risk of misaligning interventions with school needs or may lead to an imbalance of competing demands. This gap is particularly pronounced in rapidly growing urban contexts, where aspects of high enrolment, ageing infrastructure, and diverse implementation practices complicate efforts to improve sanitation management. This study therefore addressed the following research questions:

1. What domains do stakeholders prioritise in sustaining the O&M of school sanitation facilities?
2. How do these priorities differ across stakeholder groups?
3. Which specific indicators are most influential for improving sanitation service provision?

The objective of this study was to examine stakeholder-derived priorities for sustaining the O&M of school sanitation facilities in Kampala City. By comparing the perspectives of regulators, practitioners, Head Teachers, Sanitation Teachers, and researchers, the study identified areas of convergence that can strengthen coordination and areas of divergence that may undermine sustained service provision. This study focuses on school toilet facilities because they represent the primary sanitation interface for pupils and are the most sensitive to failures in O&M. Other school cleaning activities, such as classroom cleaning, compound sweeping and waste collection, fall outside the scope of this study. The findings provide evidence to guide policymakers, practitioners, and school leaders in improving governance and management of sanitation facilities and in accelerating progress towards attaining Sustainable Development Goals.

## Materials and methods

### Study area

This study was conducted in Kampala, the capital city of Uganda from July to September 2025. Kampala City has a resident population of about 1.88 million, a daytime population of 2.5 million [66], and a pupil population of about 321,000 KCCA, [34]. The city has approximately 850 primary and secondary schools with the majority privately owned (88%) and the remainder government-managed.

Sewerage coverage in Kampala is less than 15%, leaving most schools reliant on onsite sanitation systems IWA, [32]. Technologies used in Kampala schools range from flush toilets in well-resourced private schools to poorly maintained pit latrines in resource-constrained institutions. The common challenges for sanitation facilities in schools include: non-functionality, lack of privacy, inadequate handwashing facilities, limited menstrual hygiene provisions, and poor access for pupils with disabilities [13].

### Study design

This study employed a participatory multi-criteria decision analysis (MCDA) approach, using the Analytic Hierarchy Process (AHP) to derive stakeholder-informed weights for O&M domains of school sanitation facilities (T. L. Saaty, [52]; T. L. Saaty and Vargas, [55,], Tervonen, [62]. The design was guided by the need to capture the relative influence of domains that sustain sanitation service delivery without implying that any domain is dispensable. The AHP was selected because it allows complex decision problems to be structured hierarchically, integrates both qualitative judgments and quantitative scoring, and produces consistency indices to assess the reliability of stakeholder responses. This study focused specifically on the operation and maintenance of school toilet facilities and their associated service components, including cleanliness, functionality, privacy, governance, and M&E, while other school sanitation areas (such as compound/classroom cleaning), within the school environment were outside the study’s scope.

To complement the domain-level prioritisation generated through AHP and to extend the analysis to the indicator level, the SWING weighting method was applied in a complementary manner to support indicator-level weighting, given its intuitive appeal and suitability for participatory settings. Although both AHP and SWING generate weights, they serve distinct purposes in this study: AHP captures the relative importance of domains, while SWING elicits indicator-level priorities within each domain, preventing conceptual overlap between the two weighting processes. The two methods were implemented sequentially, beginning with domain weighting using AHP and followed by indicator weighting using SWING to ensure that indicator prioritisation was grounded in the relative importance of the domains. The AHP method has been extensively applied across diverse fields to determine the relative influence of factors, including health care decision-making, environmental health prioritization, and infrastructure planning [31, 75, 76]. Similarly, the SWING technique has been applied in decision-support contexts to assist modelers and decision analysts in eliciting stakeholder preferences [25, 72].

### Theoretical background of analytic hierarchy process and the SWING weighting method

The AHP, developed by Saaty [52], structures complex problems into a hierarchy of objectives, domains, and indicators. Stakeholders provide pairwise comparisons of elements using a 9-point scale, from which relative weights are derived by solving for the principal eigenvector of the comparison matrix A (Eq. 1).

1 $$\left( {A - \lambda _{{Max}} I} \right)\, \cdot \omega = 0$$

where A is the comparison matrix, I is the unit matrix, $\:{\lambda\:}_{Max}$ the largest or principal eigenvalue of vector A and $\:\omega\:$ the principal eigenvector [31, 38, 53]. The normalized eigenvector represents the relative weights of the compared elements. In this study domains were the elements, hence domain weights were determined. To ensure the logical coherence of judgments, AHP calculates a Consistency Ratio (CR) and Consistency Index (CI) (Eqs. 2 and 3).

2 $$\:\mathrm{C}\mathrm{o}\mathrm{n}\mathrm{s}\mathrm{i}\mathrm{s}\mathrm{t}\mathrm{e}\mathrm{n}\mathrm{c}\mathrm{y}\:\mathrm{I}\mathrm{n}\mathrm{d}\mathrm{e}\mathrm{x}\:\:\left(\mathrm{C}\mathrm{I}\right)=\frac{{{\uplambda\:}}_{\mathrm{M}\mathrm{a}\mathrm{x}}-\mathrm{n}}{\mathrm{n}-1}$$

3 $$\:\mathrm{C}\mathrm{o}\mathrm{n}\mathrm{s}\mathrm{i}\mathrm{s}\mathrm{t}\mathrm{e}\mathrm{n}\mathrm{c}\mathrm{y}\:\mathrm{r}\mathrm{a}\mathrm{t}\mathrm{i}\mathrm{o}\:\left(\mathrm{C}\mathrm{R}\right)\:=\frac{CR}{\mathrm{R}\mathrm{I}}$$

where n is the order of the comparison matrix and RI is the average random index derived from randomly generated matrices. RI values used in this study were adopted directly from Saaty [54] who provides the standard reference table for matrices of order 1–15. A consistency ratio (CR) of ≤ 0.1 is generally considered acceptable [52–54].

While AHP provides a structured framework with internal consistency checks, it can place a cognitive burden on participants when many comparisons are required. To address this, the SWING weighting method offers a simpler and more intuitive approach for eliciting preferences [50, 70, 72]. In this approach, participants first consider the worst-case scenario of the system (domain) and then rank the possible improvements (indicators) (“swings”) from worst to best. The most important swing is assigned 100 points, with all others scored relative to it. The swing scores are then transformed into normalized weights by dividing each individual score by the sum of all scores within the domain using Eq. 4 [35].

4 $$\:{\alpha\:}_{i}=\frac{{S}_{i}}{\sum\:_{i=1}^{k}{S}_{i}}$$

where S_i_ is the row SWING score for the indicator i and k the total number of indicator within a given domain, α_i_ is the normalized weight. This process is applied independently for each domain to ensure that indicator weights reflect the preferences specific to that domain context. SWING method is particularly valued for its simplicity, low cognitive burden, and suitability for participatory settings where stakeholders may not be familiar with complex mathematical procedures. Grounded in expected utility theory, it can be applied to explore how different factors influence the achievement of an objective [62].

### Indicator and domain selection

A structured literature review was undertaken to identify sanitation monitoring indicators applied in schools in LMICs. The search covered peer reviewed studies published between 2000 and 2025 and was supplemented with practice based program evaluations. Titles and abstracts were screened for relevance. Studies were eligible if they explicitly monitored performance, usability, or management of school sanitation facilities. Twenty four monitoring papers met the inclusion criteria. Indicators were extracted, collated, and compared to identify recurring measures across contexts.

To ensure consistency, indicator extraction followed a two-step procedure. First, all indicators reported in eligible studies were recorded using the operational descriptions provided by the study authors. Second, conceptually similar indicators were harmonised and merged to avoid duplication and to generate a consolidated indicator list suitable for school level monitoring. Each indicator was assigned a brief working definition based on source descriptions and, where available, standard terminology from Joint Monitoring Program (JMP) or WinS guidance. These indicators were then grouped into six domains, namely service planning, facility design standards, resource provision and management, service delivery, governance, and M&E (Table 1). The domain assignment was guided by established frameworks including the JMP WASH in Schools monitoring framework, the WASH in Schools (WinS) framework, the project lifecycle framework, and the Necessary Conditions for Sustainable School WASH framework [71]; WHO/UNICEF, [73]. These frameworks informed the conceptual boundaries of each domain and ensured that indicators were aligned with recognised components of sustainable sanitation service provision. Where indicators overlapped conceptually, they were merged or aligned under a common domain to avoid duplication.

**Table 1:** Indicators, domains, and framework alignment for sustaining operation and maintenance of school sanitation facilities

Indicator	Domain	Alignment with existing frameworks	References
- O&M planning	Service planning	- Project lifecycle framework - UNICEF Acceleration Framework for WASH in Schools - JMP WASH in Schools monitoring framework	[3, 10, 12, 13, 19, 45, 46, 60, 67]
- Clear roles and responsibilities			
- Dedicated budget-line			
- Preventive maintenance schedule			
- Sanitation technology	Facility design standards	- WinS framework - JMP WASH in Schools sanitation service ladders	[3, 5, 6, 9, 10, 12, 13, 14, 15, 16, 19, 24, 27, 28, 29, 30, 33, 39, 45, 46, 47, 48, 56, 58, 60, 61, 69]
- Safe containment of excreta			
- Facility accessibility for people with disability			
- Sex segregation			
- Privacy			
- Menstrual hygiene management (MHM) facility			
- Handwashing facilities			
- Pupil to stance ratio			
- Trained personnel	Resources management	- Necessary Conditions for Sustainable School WASH framework - UNICEF Acceleration Framework for WASH in Schools	[5, 15, 16, 19, 45, 46, 47, 60]
- Reliable water supply			
- Budget-line implementation			
- Material-use tracking system			
- Facility availability	Service delivery	- WinS framework - JMP WASH in Schools service ladders	[1, 2, 3, 5, 6, 9, 10, 12, 15, 16, 19, 23, 24, 28–30, 33, 39, 45–47, 56, 58, 60]
- General facility accessibility			
- Facility functionality			
- Toilet cleanliness			
- Toilet consumables at point of use			
- MHM materials availability			
- Safe disposal of solid waste			
- Safe excreta disposal			
- Hygiene promotion activities	Governance	- Citywide Inclusive Sanitation framework - Sanitation and Water for All building blocks - Necessary Conditions for Sustainable School WASH framework	[2, 16, 19, 24, 46, 60]
- Formal school management structures			
- Internal and external stakeholder engagement			
- Enforcement			
- Use of structured M&E tools	M&E	- Necessary Conditions for Sustainable School WASH framework - UNICEF Acceleration Framework for WASH in Schools - JMP WASH in Schools monitoring framework	[19, 27, 47, 60]
- Multiple stakeholder monitoring			
- Making informed decisions from monitoring data			
- M&E training of personnel			
- Escalation process for unresolved issues			

Indicators presented in this table reflect harmonised definitions developed during the indicator extraction process; conceptually similar items were merged to avoidduplication

### Workshop participant selection

Stakeholders were purposively selected to ensure balanced perspectives across five categories: regulators, researchers, practitioners, Head Teachers and Sanitation Teachers. The selection criteria required participants to have direct experience with school sanitation: management, programme implementation, regulation, or research. Seven participants were recruited per stakeholder category to balance breadth of perspectives across the five roles with the cognitive demands of AHP and SWING elicitation, consistent with MCDA good practice [40]. The regulators category comprised of inspectors and sanitation engineers from the Ministry of Health, Ministry of Water and Environment, Ministry of Education and Sports and KCCA. Researchers were drawn from universities within Kampala and had published work on school sanitation. Practitioners were selected from civil society organizations implementing water, sanitation, and hygiene (WASH) projects in schools. The selection of head teachers and sanitation teachers ensured coverage of primary and secondary schools, public and private institutions, and each of the city’s five administrative divisions.

### Data collection instruments and procedures

Data collection employed questionnaires designed for the SWING and AHP methods (Supplementary 3). The questionnaires underwent expert review and pre-testing to ensure clarity and appropriateness for the different respondent groups. The SWING questionnaire required participants to assign relative weights to indicators within each domain in terms of their influence on achieving the domain objective. The AHP questionnaire on the other hand required participants to rate the relative influence of domains in achieving the overarching objective of sustaining O&M of school sanitation facilities. The validity of the data collection instruments was addressed by adapting established MCDA instruments, subjecting them to expert review to ensure content relevance, and pre-testing them to confirm clarity and contextual appropriateness prior to data collection.

During the workshop, upon registration and signing consent forms, participants were grouped by stakeholder category and each group was assisted by a trained Research Assistant. Following a briefing on the objectives and methods, participants completed SWING weighting tables for indicators and then carried out AHP pairwise comparisons of domains using Saaty’s 9-point scale [52]. Consistency ratios were checked immediately, and any comparison exceeding a CR value of 0.1 was repeated to improve reliability. All AHP and SWING judgments were completed individually.

### Data analysis

Pairwise comparison matrices from each participant were analysed using standard AHP procedures [51]. Individual judgments were aggregated within stakeholder groups using the geometric mean, and overall domain weights were subsequently computed across all participants. Reliability of stakeholder judgements was assessed using AHP consistency ratio thresholds (CR ≤ 0.1), with only consistent responses retained for analysis. Uncertainty in domain weights was estimated using bootstrap resampling (10,000 iterations) to generate 95% confidence intervals (CI). Differences between stakeholder groups were assessed using the Kruskal–Wallis test, which showed no statistically significant variation in domain weights. Local indicator weights were by normalizing the averaged SWING scores form all participants. Global indicator weights were then calculated as the product of domain weights and local indicator weights, enabling direct comparison of indicator importance across the entire framework. Reliability of the SWING results was supported through the use of a standardised elicitation procedure and comparisons of indicator rankings across stakeholder groups to examine patterns of convergence and divergence.

## Results and discussion

### Participant characteristics

A total of 35 participants took part in the weighting exercise, representing 13 different professions with mean work experience of over ten years across all stakeholder groups (Table 2). Researchers had the highest mean work experience of 24 years, while Sanitation Teachers had the lowest at 12 years. Educational qualifications ranged from Grade III certificates to doctoral degrees, which meant that the perspectives of frontline implementers, practitioners, and academic or policy experts were all represented.

**Table 2:** Professional and educational characteristics of stakeholder groups

Stakeholder group	Professions	Mean experience (years)	Education qualification range
Regulators	Teachers; Civil Engineers; Environmental Health Officers	14	Bachelor’s to Master’s degree
Researchers	Civil Engineering; Environmental Engineering; Environmental Management	24	Doctoral degree
Practitioners	Accounting; Information Technology; Social Sciences; Social Work and Social Administration; Bio-systems Engineering; Environmental Health Science	13	Bachelor’s to Master’s degree
Head Teachers	Teachers (primary or secondary schools)	22	Bachelor’s to Master’s degree
Sanitation Teachers	Teachers (primary or secondary schools)	12	Grade III Certificate to Bachelor’s degree

The diversity of participants in terms of profession, area of work, and years of experience as shown in Table 2, strengthens legitimacy of the participatory decision-support tools by balancing technical rigor with contextual relevance [12, 26, 54]. The variation in academic qualifications further highlights the value of incorporating both academic and practical expertise, consistent with recommendations for inclusive stakeholder engagement in the WASH and education sectors [64, 65]. This diversity also enhances the reliability of the weighting exercise by ensuring that the resulting priorities reflect not only technical accuracy but also user-centered and context-specific considerations.

### AHP domain weights

**Fig. 1:**
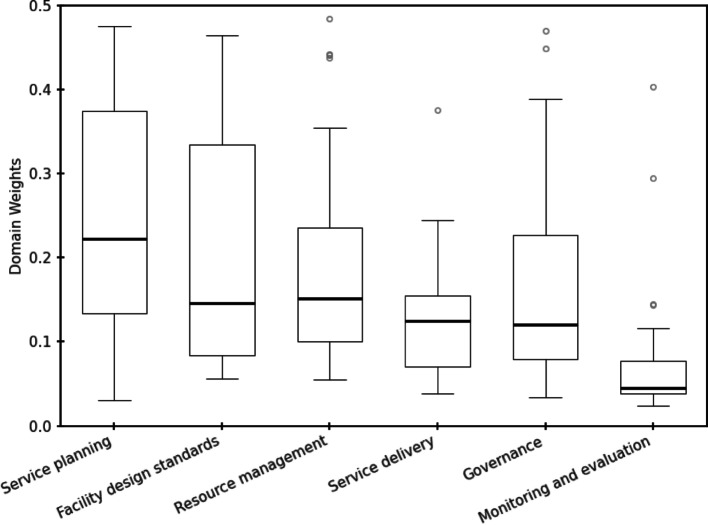
Distribution of AHP-derived weights for domains of school sanitation operation and maintenance

The AHP results indicated variation in the relative importance assigned to the six domains of O&M for school sanitation (Fig. 1 and Supplementary 1). Service planning received the highest overall weight (0.234, CI 0.183–0.289), followed by resource management (0.199, CI 0.162–0.241) and facility design standards (0.193, CI 0.151–0.241), while M&E consistently had the lowest weight (0.071, CI 0.056–0.090). With the exception of service planning and M&E, the weights of the remaining domains were relatively close to each other (less than one unit weight).

The prominence of service planning, facility design standards, and resource management as shown in Fig. 1, highlights their foundational role in sustaining O&M for school sanitation. This finding aligns with Pu et al., [49], who emphasized resources, information, and accountability as critical conditions for sustainable sanitation services. Planning defines responsibilities, clarifies budgets, and supports proactive maintenance, while facility design standards ensure inclusivity, resilience, and compliance with minimum benchmarks. Similarly, Byansi et al., [13] demonstrated that dedicated sanitation budgets and adequate facility maintenance improve service levels, underscoring the centrality of resource-based support.

The consistently low weights assigned to M&E domain can be interpreted in light of constraints experienced by both implementing organisations and school level actors. Deroo et al., [22] found that organisations supporting WASH in schools often faced limited staff capacity, constrained funding, logistical challenges, and inadequate management systems, factors that weaken the regularity and usefulness of monitoring activities. Practitioners involved in this study may therefore draw on these experiences when prioritising domains. At school level, Snel et al., [59] observed that M&E is rarely undertaken systematically because of insufficient staff training and weak follow up mechanisms. Teachers and Head Teachers already carry substantial instructional and administrative responsibilities, making it difficult to integrate additional monitoring tasks into existing workloads [15]. Schools also lack customised monitoring tools suited to daily operational realities, further reducing the feasibility of routine monitoring. This low prioritisation may perpetuate weak accountability even when planning and budgeting functions are strengthened.

The relatively close weights assigned to facility design standards, resource management, service delivery, and governance reflect their character as interdependent, day-to-day functions necessary for the smooth O&M. Stakeholders therefore regarded these domains as equally critical for sustaining operational continuity, even though none carried the same foundational weight as planning.

### Domain weight variation across experts groups

**Fig. 2:**
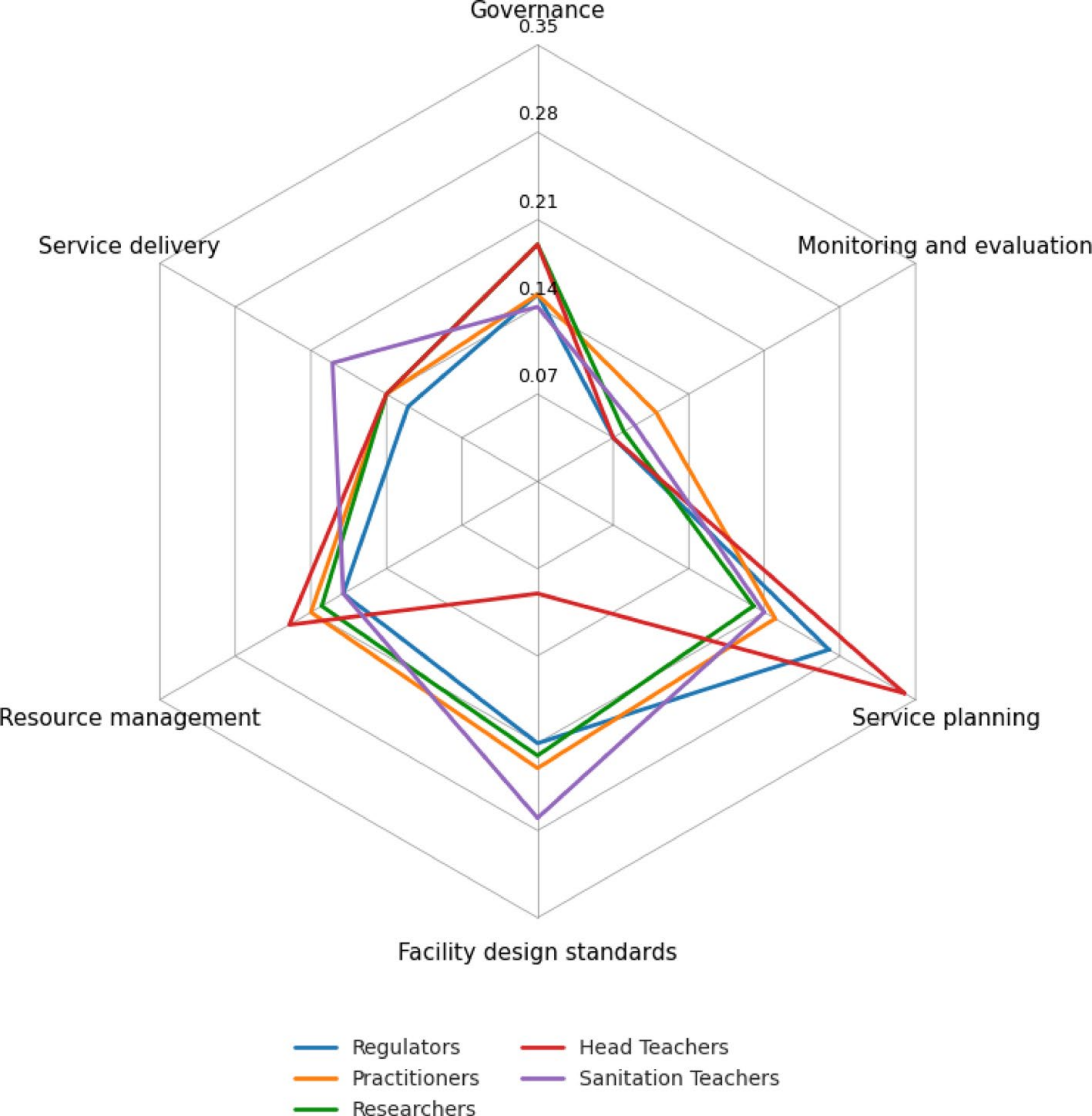
Domain weights for school sanitation operation and maintenance by the stakeholder groups

The distribution of weights across expert groups showed broad convergence in recognizing service planning, facility design standards, and resource management as the most influential domains for sustaining sanitation services in schools (Fig. 2). Head Teachers emphasized service planning, resource management, and governance, while Sanitation Teachers assigned higher weights to facility design standards and service delivery. Practitioners placed greater emphasis on M&E, whereas researchers and regulators showed a balanced distribution across domains, with both tending to assign higher weight to facility design standards.

The subtle differences in domain weighting across stakeholder groups as seen in Fig. 2, reveal how institutional roles shape priorities for sanitation O&M. Head Teachers emphasized service planning, resource management, and governance. This emphasis is consistent with their statutory duties under the The Education (Pre-Primary, Primary and Post-Primary) Act, (2008) [63], which assign them responsibility for administering school funds, preparing annual plans and budgets, and supervising school activities. In practice, these duties necessitate planning and systems for efficient resource use at school level, which explains the observed weighting pattern. Furthermore, this finding aligns with Kim et al., [36] and Sariakin et al., [57] who showed that leadership and management practices are central to maintaining continuity and accountability in school settings.

Sanitation Teachers assigned higher weights to facility design standards and service delivery. This is in line with Appiah-Brempong et al., [8] and McMichael [42], who reported that frontline staff often prioritize usability and cleanliness as immediate determinants of pupil wellbeing. Practitioners placed relatively greater emphasis on M&E, echoing evidence from Deroo et al., [22] that program implementers depend on performance tracking and data systems to strengthen service delivery.

The balanced distribution among researchers and regulators, with slightly higher weighting for facility design standards, is consistent with Morgan et al., [44], who emphasized compliance and adherence to technical benchmarks as essential for sustaining service provision. Although numerical differences across stakeholder groups were not statistically significant, the patterns reflect distinct operational realities. Regulators tended to prioritise governance and planning in line with their oversight mandates, while practitioners placed greater weight on service delivery and resource management due to their implementation roles under constrained budgets.

School-level actors, particularly Head Teachers and Sanitation Teachers, focused more on daily functionality and facility conditions, reflecting the immediate pressures of ensuring pupil safety and usability. These role-specific tendencies indicate that successful implementation will depend on providing guidance and support that are adapted to the operational capacities of each stakeholder group. Collectively, these patterns suggest that while stakeholder priorities differ subtly according to their roles, implementation strategies must account for these differences while leveraging shared priorities to sustain sanitation services in schools.

### Proposed model for implementation of sustainable operations and maintenance of school sanitation facilities

The application of the SWING method produced the local weights of the indicators (Supplementary 2), which were then combined with the domain weights to generate global weights presented in Table 3. These global weights highlight the indicators with the greatest influence on sustainable O&M of school sanitation. The top five were the presence of an O&M plan (0.067), a dedicated budget line (0.067), clear roles and responsibilities (0.059), budget-line implementation (0.056), and reliable water supply (0.054). Notably, four of these five indicators are directly linked to planning and resources, underscoring their dominant role in sustaining services. In contrast, the five least weighted indicators were the escalation process for unresolved issues (0.011), safe disposal of solid waste (0.013), making informed decisions from monitoring data (0.014), availability of MHM materials (0.014), and safe disposal of excreta (0.017). All indicators directly linked to M&E fell within the bottom quartile of weights, reflecting the current undervaluation of accountability and feedback mechanisms in schools.

**Table 3:** Proposed hierarchical model for sustainable operation and maintenance of school sanitation facilities

Domain	Domain weight	Indicator code	Indicator	Local weight	Global weight
Service planning	0.234	W1.1	O&M plan	0.287	0.067
		W1.2	Clear roles and responsibilities	0.254	0.059
		W1.3	Dedicated budget line	0.287	0.067
		W1.4	Preventive maintenance schedule	0.172	0.040
Facility design standards	0.193	W2.1	Sanitation technology	0.151	0.029
		W2.2	Safe containment of excreta	0.159	0.031
		W2.3	Facility accessibility for people with disability	0.105	0.020
		W2.4	Sex segregation	0.132	0.025
		W2.5	Privacy	0.123	0.024
		W2.6	MHM facility availability	0.105	0.020
		W2.7	Handwashing facilities	0.119	0.023
		W2.8	Pupil to stance ratio	0.106	0.020
Resource management	0.199	W3.1	Trained personnel	0.231	0.046
		W3.2	Reliable water supply	0.269	0.054
		W3.3	Budget line implementation	0.282	0.056
		W3.4	Material-use tracking system	0.218	0.043
Service delivery	0.137	W4.1	Facility availability	0.148	0.020
		W4.2	General facility accessibility	0.146	0.020
		W4.3	Facility functionality	0.149	0.020
		W4.4	Toilet cleanliness	0.130	0.018
		W4.5	Toilet consumables at point of use	0.117	0.016
		W4.6	MHM materials availability	0.103	0.014
		W4.7	Safe disposal of solid waste	0.092	0.013
		W4.8	Safe excreta disposal	0.115	0.017
Governance	0.166	W5.1	Hygiene promotion activities	0.246	0.041
		W5.2	Formal school management structures	0.278	0.047
		W5.3	Internal and external stakeholder engagement	0.218	0.036
		W5.4	Enforcement	0.258	0.043
M&E	0.071	W6.1	Use of structured M&E tools	0.230	0.016
		W6.2	Multiple stakeholder monitoring	0.197	0.014
		W6.3	Making informed decisions from monitoring data	0.196	0.014
		W6.4	M&E training for personnel	0.220	0.016
		W6.5	Escalation process for unresolved issues	0.157	0.011

The five most highly weighted indicators shown in Table 3, underscore the importance of management- and resource-oriented levers in sustaining O&M. This aligns with field evidence from Kampala, where schools with dedicated sanitation budgets and stronger O&M practices were significantly more likely to reach basic service levels [13]. Furthermore, this finding is supported by Alexander et al., [4], who showed that recurrent operational costs are essential to maintaining functional school WASH, and by McGinnis et al., [41], who identified inadequate financing and budgeting as key barriers to sustaining services in educational settings. The prioritization of clear roles and responsibilities in this study also resonates with Valcourt et al., [68], who noted that weak accountability structures frequently undermine WASH system performance. Reliable water supply, another top-ranked indicator, reflects a foundational requirement for both hygiene practices and facility functionality, consistent with Appiah-Brempong et al., [8], who found that schools with financial provisions for water supply were far more likely to maintain functional handwashing stations. Collectively, these results suggest that systematic planning, adequate and implemented financing, and reliable inputs, reinforced by role clarity, are critical for sustaining sanitation services in schools.

In contrast, the least weighted indicators highlight the persistent undervaluation of accountability, waste management, and user-centered service elements. Deroo et al., [22] reported similar challenges, observing limited monitoring capacity and missed opportunities to use evaluation data to improve programs, while Appiah-Brempong et al., [8] documented gaps in menstrual hygiene resources that restrict the inclusivity of sanitation provision. Although monitoring-related indicators received the lowest weights, their role in providing feedback and supporting adaptive management remains essential for long-term sustainability. The low ranking of these indicators suggests that a gap may emerge between what stakeholders value and what schools can realistically implement, particularly where financial, administrative, or technical constraints limit routine O&M activities. This underscores the need to strengthen monitoring and accountability systems in a way that does not impose additional administrative burden on already overstretched school staff.

Implementing these priorities may be challenging for schools with limited resources, particularly those that operate with constrained budgets, few administrative staff, and competing instructional responsibilities. Ensuring feasibility will require simple tools, clear expectations, and supportive supervision to enable schools, especially those facing resource constraints, to adopt and apply the highest weighted indicators.

### Study limitations

The AHP and SWING methods rely on structured stakeholder judgements which, although systematically elicited, remain subjective and may be influenced by participants’ professional backgrounds. Concurrent field observation or facility level data were not collected during the weighting period, which limited the ability to triangulate stakeholder derived priorities with actual O&M performance. The cross sectional design also restricts inference about how priorities may shift over time or in response to changes in school conditions. In addition, the study did not explicitly integrate broader equity dimensions such as gender and disability, and differences in weighting across stakeholder subgroups were not examined. Furthermore, because the stakeholder sample was drawn from urban Kampala, the findings may not be generalizable to rural schools or other regions with different resource constraints or management structures.

## Conclusions

The results of this study show that stakeholders prioritize management focused domains for sustained sanitation services, with service planning, facility design standards, and resource management emerging as the top priorities. Indicators such as the presence of an O&M plan, a dedicated and implemented budget line, reliable water supply, and clear role allocation were ranked highest, underscoring the importance of planning and predictable resourcing. Although M&E indicators consistently received the lowest weights, their value for feedback and adaptive management remains critical.

Across regulators, practitioners, Head Teachers, Sanitation Teachers, and researchers, no statistically significant differences were observed in domain weights, demonstrating broad convergence with only minor variations in emphasis. The highest ranked indicators across all groups, including the O&M plan, the dedicated budget line, role clarity, budget execution, and reliable water supply, represent the levers considered most influential for improving sustained sanitation service provision. Taken together, these findings provide a stakeholder informed evidence base to strengthen planning systems, ensure reliable resource flows, and support progress toward sustainable sanitation service provision in schools.

## Policy, practice and research implication of this study

The findings provide direction for education and health policymakers in allocating resources to sustain school sanitation O&M. The prominence of planning, dedicated budget lines, and accountability indicators highlights the need for policies that require schools to prepare operational plans, define responsibilities, and secure predictable financing. Embedding these within sector planning and inspection frameworks would strengthen accountability and help ensure functional and sustainable sanitation facilities. Given the consistently low prioritization of M&E, policies should also support the development of simple, context specific monitoring tools, set clear reporting expectations, and integrate sanitation indicators into routine school inspection systems to enhance follow up and strengthen accountability.

For practitioners, including Head Teachers and Sanitation Teachers, the results highlight actionable priorities such as ensuring reliable water supply, implementing budgets, and tracking material use. Strengthening school level capacity for routine monitoring, including training and practical tools, can support more consistent oversight of functionality and cleanliness. These actions can improve daily management, support preventive maintenance, and enhance pupil wellbeing.

This study also contributes to the evidence on sustainable school sanitation by presenting a stakeholder informed hierarchy of domains and indicators. Future research should validate the model through implementation in diverse contexts including rural, peri-urban, and informal urban settings to assess its transferability and external validity. Such validation should include concurrent field measurements to examine whether improvements in the highest weighted indicators translate into sustained gains in service levels. Because operation and maintenance needs and stakeholder priorities may evolve over time, periodic reassessment is recommended to ensure continued relevance and responsiveness to changing operational realities. Future studies should also examine how governance structures, resource constraints, and implementation capacity influence prioritisation patterns across regions, together with equity sensitive weighting approaches that incorporate gender and disability perspectives.

Finally, future applications of the model should link improvements in key indicators such as functionality, cleanliness, and reliable water supply with health and educational outcomes including diarrhoeal disease incidence, absence related to menstrual hygiene challenges, and overall school attendance. Demonstrating these links will help quantify the public health and educational impact of strengthened operation and maintenance.

## Supplementary Information 

Supplementary Material 1.

Supplementary Material 2.

Supplementary Material 3.

## Data Availability

The datasets generated during and/or analysed during the current study are available in the Zenodo repository: https://doi.org/10.5281/zenodo.17812613.

## Abbreviations

AHP: Analytic Hierarchy Process
CI: Consistency Index
CR: Consistency Ratio
JMP: Joint Monitoring Programme
IWA: International Water Association
KCCA: Kampala Capital City Authority
LMICs: Low- and Middle-Income Countries
MCDA: Multi-Criteria Decision Analysis
M&E: Monitoring and Evaluation
MHM: Menstrual Hygiene Management
O&M: Operation and Maintenance
UBOS: Uganda Bureau of Statistics
UNCST: Uganda National Council for Science and Technology
UNICEF: United Nations Children's Fund
WASH: Water Sanitation and Hygiene
WHO: World Health Organization
WinS: Water, Sanitation and Hygiene in Schools
