# Supplementary material for: Stakeholder priorities for sustaining operations and maintenance of school sanitation facilities in Kampala City, Uganda
Source: Discov Public Health. 2026 May 29;23(1):795. doi: 10.1186/s12982-026-02148-x (PMC13221315; doi:10.1186/s12982-026-02148-x)
Supplement: Supplementary file 1 — Supplementary Material 1. [file 12982_2026_2148_MOESM1_ESM.docx]

**SUPPLEMENTARY 1**

**Table 4. AHP Weights and Statistical Comparison of O&M Domains**

| **Domain** | **Overall weight** | **CI-Low** | **CI-High** | **Kruskal–Wallis** | **p-value** |
| --- | --- | --- | --- | --- | --- |
| Service planning | 0.234 | 0.183 | 0.289 | 6.216 | 0.184 |
| Facility design standards | 0.193 | 0.151 | 0.241 | 8.804 | 0.066 |
| Resource management | 0.199 | 0.162 | 0.214 | 2.169 | 0.705 |
| Service delivery | 0.137 | 0.111 | 0.166 | 3.353 | 0.501 |
| Governance | 0.166 | 0.129 | 0.208 | 4.777 | 0.311 |
| Monitoring and evaluation | 0.071 | 0.056 | 0.09 | 1.264 | 0.867 |
