# Supplementary material for: Stakeholder priorities for sustaining operations and maintenance of school sanitation facilities in Kampala City, Uganda
Source: Discov Public Health. 2026 May 29;23(1):795. doi: 10.1186/s12982-026-02148-x (PMC13221315; doi:10.1186/s12982-026-02148-x)
Supplement: Supplementary file 2 — Supplementary Material 2. [file 12982_2026_2148_MOESM2_ESM.docx]

**SUPPLEMENTARY 2**


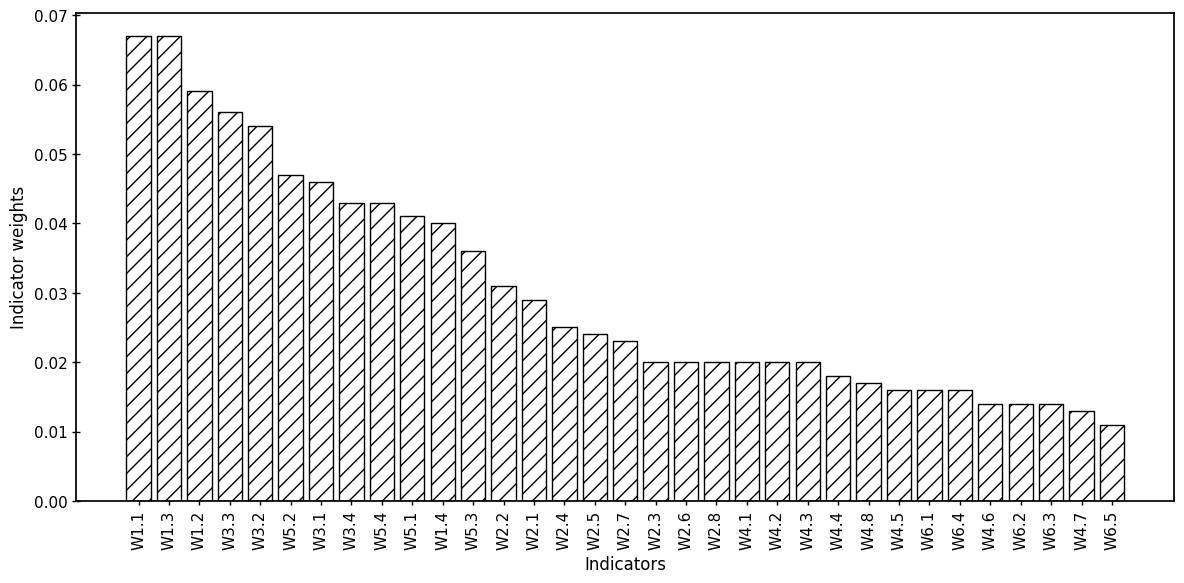


**Figure 3. Global Weights of Indicators for Sustainable O&M of School Sanitation Facilities**
