# Supplementary material for: Stakeholder priorities for sustaining operations and maintenance of school sanitation facilities in Kampala City, Uganda
Source: Discov Public Health. 2026 May 29;23(1):795. doi: 10.1186/s12982-026-02148-x (PMC13221315; doi:10.1186/s12982-026-02148-x)
Supplement: Supplementary file 3 — Supplementary Material 3. [file 12982_2026_2148_MOESM3_ESM.docx]

**SUPPLEMENTARY 3**

**DESIGN WORKSHOP FOR DETERMINATION OF REALTIVE IMPORTANCE OF DOMAINS FOR SUSTAINABLE OPERATION AND MAINTENANCE (O&M) OF SANITATION FACILITIES IN SCHOOLS**

**QUESTIONNAIRE 1: OPERATION AND MAINTENANCE INDICATOR WEIGHTING USING SWING METHOD**

**Question:** Using SWING decision support tool to determine the normalized weights of indicators in each domain

**Objective:** To determine the relative importance of operation and maintenance indicators in achieving the domain requirements **Procedure:**

For a given domain;

1. assume that all the indicators given are at the worst possible state.
2. "Swing" each indicator from worst to best.
3. Identify the indicator that provides the greatest value when improved from worst to best. Score that indicator by 100 points
4. Score the rest of the indicators relative to their improvements using expert judgement
5. Allocate scores of the indicator in consideration based on how desirable or valuable that swing is.
6. A consensus must be reached from the group during the scoring session
7. For each domain normalize the scores to a scale of 100% to obtain each indicator score.

Domain **1.0: Service Planning** – Establishment of formal, documented, and forward-looking arrangements for the operation and maintenance of sanitation facilities, including plans, assigned responsibilities, budgets, and preventive schedules.

|  | **Indicator** | **Rank** | **Relative score**  **(points)** | **Normalized weight score (%)** |
| --- | --- | --- | --- | --- |
| 1.1 | School having a **documented O&M plan** |  |  |  |
| 1.2 | Cleary documented **roles and responsibilities** for O&M of sanitation facilities |  |  |  |
| 1.3 | School with **dedicated budget-line** for sanitation in the school’s term or annual budget |  |  |  |
| 1.4 | School with **documented preventive maintenance schedule** for sanitation facilities (inside or outside the O&M plan) |  |  |  |
|  | Total |  |  |  |

Domain **2.0: Facility Design Compliance and Suitability** – Adherence of sanitation facilities to national and JMP design standards, and provision of infrastructure that is safe, inclusive, and appropriate for all intended users.

|  | **Indicator** | **Rank** | **Relative score**  **(points)** | **Normalized weight score (%)** |
| --- | --- | --- | --- | --- |
| 2.1 | **Type of sanitation technology in use (**Improved / Unimproved) |  |  |  |
| 2.2 | **Sanitation technology providing safe containment of excreta** |  |  |  |
| 2.3 | **Toilet facilities accessibility** to persons with disabilities |  |  |  |
| 2.4 | **Toilet sex segregation** |  |  |  |
| 2.5 | **Toilet privacy** |  |  |  |
| 2.6 | **Provision of menstrual hygiene management (MHM) space** with water, soap, and disposal facilities |  |  |  |
| 2.7 | Provision of **functional handwashing facilities** with water and soap |  |  |  |
| 2.8 | Meeting **Pupil-to-stance ratio** standards |  |  |  |
|  | **Total** |  |  |  |

Domain **3.0: Resources and Resources-Management systems for O&M** – Availability, organization, and utilization of financial, human, material, and water resources necessary to sustain sanitation operation and maintenance.

|  | **Indicator** | **Rank** | **Relative score**  **(points)** | **Normalized weight score (%)** |
| --- | --- | --- | --- | --- |
| 3.1 | **Use of trained sanitation O&M personnel** |  |  |  |
| 3.2 | **Reliable and sufficient water supply** |  |  |  |
| 3.3 | **Implementation of sanitation budget line** |  |  |  |
| 3.4 | **Material supply and use tracking system** |  |  |  |
|  | **Total** |  |  |  |

Domain **4.0: Service Delivery and Maintenance** – Day-to-day operation of sanitation facilities, ensuring they are available, accessible, functional, clean, supplied, gender-sensitive, and supported by safe waste disposal practices.

|  | **Indicator** | **Rank** | **Relative score (points)** | **Normalized weight score (%)** |
| --- | --- | --- | --- | --- |
| 4.1 | **Toilet facility availability** (not reserved exclusively for special groups) |  |  |  |
| 4.2 | **Toilet facility operational accessibility** all the time |  |  |  |
| 4.3 | **Facility functionality** |  |  |  |
| 4.4 | **Toilet cleanliness** |  |  |  |
| 4.5 | **Availability of consumables at point of use** |  |  |  |
| 4.6 | **Maintenance and stocking of MHM facilities** |  |  |  |
| 4.7 | **Safe disposal of solid waste** |  |  |  |
| 4.8 | **Safe disposal of excreta or fecal sludge** |  |  |  |
|  | **Total** |  |  |  |

Domain **5.0: Institutional Management and Engagement** – School leadership, governance structures, policies, and stakeholder participation mechanisms that support and sustain sanitation O&M.

|  | **Indicator** | **Rank** | **Relative score (points)** | **Normalized weight score (%)** |
| --- | --- | --- | --- | --- |
| 5.1 | **Promotional activities for sanitation and hygiene knowledge** |  |  |  |
| 5.2 | **Presence of formal school governance structures** (e.g., sanitation committee, focal teacher) |  |  |  |
| 5.3 | **Active engagement of internal and external stakeholders** (SMC, PTA, pupils, community, NGOs, local authorities) |  |  |  |
| 5.4 | **Existence and enforcement of school sanitation rules** or guidelines |  |  |  |
|  | **Total** |  |  |  |

Domain **6.0: Monitoring and Inspection** – Structured, regular, and documented assessment of sanitation facilities, involving multiple stakeholders, with findings used for decision-making, escalation, and improvement.

|  | **Indicator** | **Rank** | **Relative score (points)** | **Normalized weight score (%)** |
| --- | --- | --- | --- | --- |
| 6.1 | Use of structured tools for **sanitation monitoring** |  |  |  |
| 6.2 | Involvement of **multiple stakeholders** in Sanitation monitoring |  |  |  |
| 6.3 | Use of **monitoring data for informed decision making** |  |  |  |
| 6.4 | Training of sanitation personnel on sanitation monitoring and inspection methods |  |  |  |
| 6.5 | D**ocumented process for escalating** unresolved sanitation issues |  |  |  |
|  | **Total** |  |  |  |

**QUESTIONNAIRE 2: OPERATION AND MAINTENANCE DOMAIN WEIGHTING USING ANALYTIC HIERARCHY PROCESS (IAHP) METHOD**

1. **EXERCISE 2:** Use of Analytic Hierarchy Process (IAHP) to obtain domain weights for the operation and maintenance of sanitation facilities in schools
2. **OBJECTIVE:** To ensure that sanitation facilities in schools are operated and maintained sustainably to provide safe, reliable, and adequate services.
3. **CRITERIA** (what “matters” when judging the domains): This governed by your experience. However, Table 1 provides some of the issues that can guide you to bring out your experience.

**Table1: AHP Criteria and Considerations for Pairwise Comparison of O&M Domains**

| **Criteria (area of consideration)** | **Key Question to Ask About the Domain** | **Examples of Issues to Think About** |
| --- | --- | --- |
| **Health Protection** | Does the domain reduce the risk of disease transmission and ensure safe hygiene for all users? | Pathogen reduction, safe disposal of waste, hygiene promotion. |
| **Dignified and Acceptable Service Provision** | Does the domain ensure facilities are adequate, private, sex-segregated, culturally appropriate, inclusive, and acceptable to all intended users? | Privacy, gender equity, disability inclusion, cultural fit, user comfort, willingness to use facilities. |
| **Environmental Protection** | Does the domain prevent environmental contamination and protect surrounding water, soil, and air quality? | Safe waste management, pollution prevention, sustainable disposal methods. |
| **Reliability and Continuity** | Does the domain ensure facilities remain functional and accessible over time, through consistent stakeholder participation in operation and maintenance? | Operational uptime, timely repairs, preventive maintenance, stakeholder engagement in upkeep. |

1. **ALTERNATIVES** (the things you are prioritizing): Domains
2. **Service Planning: Planning**
3. **Facility Design Compliance and Suitability: Standards**
4. **Resource Mobilization and Management: Resources**
5. **Service Operation and Maintenance: Sanitation service access**
6. **Institutional Support and Participation: Governance**
7. **Monitoring, Inspection, and Feedback: M&E**
8. **EXAMPLE.**
9. Consider the distribution of a service with domains A – F. The worst case scenario is when all domains score 0, the bars in the graph indicate the influences on the system if a domain is implemented.


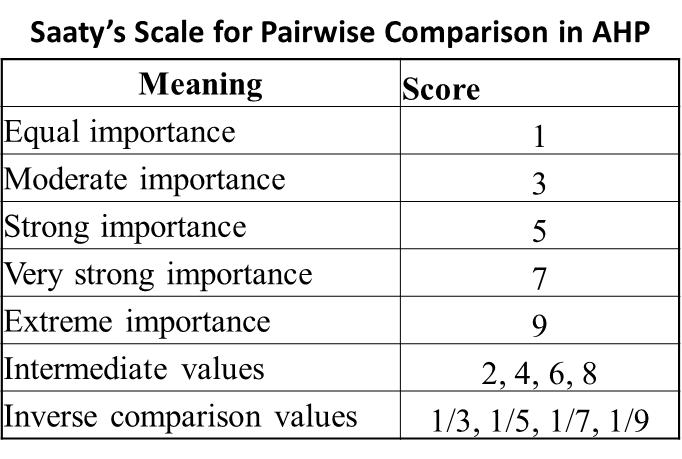


1. Scoring process

| **Pairwise comparison** | **Level of influence** | **Score** |
| --- | --- | --- |
| A and A | Equal importance | 1 |
| A and B | Moderate importance | 3 |
| A and C | Strong Importance | 5 |
| A and E | Very strong Importance | 7 |
| A and F | Extremely Important | 9 |
| B and C | Strong Importance | 5 |
| B and D | Moderate importance | 3 |
| D and E | Very strong Importance | 7 |
| D and F | Extremely Important | 9 |
| C and F | Very strong Importance | 7 |
| C and E | Moderate importance | 3 |

1. Example of scoring

|  | A | B | C | D | E | F |
| --- | --- | --- | --- | --- | --- | --- |
| A | 1 | 3 | 5 | 3 | 7 | 9 |
| B | 0.33333333 | 1 | 5 | 1 | 5 | 9 |
| C | 0.2 | 0.2 | 1 | 0.333333 | 3 | 7 |
| D | 0.33333333 | 1 | 3 | 1 | 5 | 9 |
| E | 0.14285714 | 0.2 | 0.333333 | 0.2 | 1 | 5 |
| F | 0.11111111 | 0.1111111 | 0.142857 | 0.111111 | 0.2 | 1 |

**Consistency level : 0.076173222 < 0.1**

1. **PROCEDURE**
2. Rank the alternatives in consideration of the importance towards achieving the objective in the schools setting.

**OBJECTIVE:** To ensure that sanitation facilities in schools are operated and maintained **sustainably** to provide **safe, reliable, and adequate** services.

1. Using the SWING method

Fill in the table below.

| **Domain** | First time Ranking) | | Second time Ranking | | Final ranking | |
| --- | --- | --- | --- | --- | --- | --- |
|  | Rank | scores | Rank | scores | Rank | scores |
| 1. **Planning** |  |  |  |  |  |  |
| 1. **Standards** |  |  |  |  |  |  |
| 1. **Resources** |  |  |  |  |  |  |
| 1. **Sanitation service access** |  |  |  |  |  |  |
| 1. **Governance** |  |  |  |  |  |  |
| 1. **Monitoring and evaluation** |  |  |  |  |  |  |

1. Complete the domain comparison matrix on your worksheet
2. While completing the matrix, ensure consistency.
3. Use consensus decision-making — discuss and agree as a group
4. Your scores will be used to generate domain weights in the DSS. Ensure consistency.

| **Compare →vs ↓** | **Service planning** | **Facility design compliance and suitability** | **Resources management** | **Service Delivery and Maintenance** | **Institutional Management and Engagement** | **Monitoring and Inspection** |
| --- | --- | --- | --- | --- | --- | --- |
| **Service planning** | **1** |  |  |  |  |  |
| **Facility design compliance and suitability** |  | **1** |  |  |  |  |
| **Resources management** |  |  | **1** |  |  |  |
| **Service Delivery and Maintenance** |  |  |  | **1** |  |  |
| **Institutional Management and Engagement (Governance)** |  |  |  |  | **1** |  |
| **Monitoring and Inspection** |  |  |  |  |  | **1** |

1. Check for consistency (facilitators to do this)
2. Determine the weights (facilitators to do this)
3. Submit to your results when consistence ratio is less than 0.1
